# Statement of Retraction: Methyl-CpG-binding protein 2 promotes osteogenic differentiation of bone marrow mesenchymal stem cells through regulating forkhead box F1/Wnt/β-Catenin axis

**DOI:** 10.1080/21655979.2023.2269350

**Published:** 2023-10-18

We, the Editors and Publisher of the journal *Bioengineered*, have retracted the following article:

Weiqin Ji & Xiaotong Sun (2022) Methyl-CpG-binding protein 2 promotes osteogenic differentiation of bone marrow mesenchymal stem cells through regulating forkhead box F1/Wnt/β-Catenin axis, Bioengineered, 13:1, 583-592, DOI: 10.1080/21655979.2021.2012357

Since publication, significant concerns have been raised about the integrity of the data and reported results in the article. When approached for an explanation, the authors did not respond and have not provided their original data or any necessary supporting information.

As verifying the validity of published work is core to the integrity of the scholarly record, we are therefore retracting the article. The corresponding author listed in this publication has been informed.

We would welcome an explanation from the authors at any point. We have been informed in our decision-making by our editorial policies and the COPE guidelines.

The retracted article will remain online to maintain the scholarly record, but it will be digitally watermarked on each page as ‘Retracted’.

